# Bacterial Community Spacing Is Mainly Shaped by Unique Species in the Subalpine Natural Lakes of China

**DOI:** 10.3389/fmicb.2021.669131

**Published:** 2021-07-01

**Authors:** Jinxian Liu, Jiahe Su, Meiting Zhang, Zhengming Luo, Xiaoqi Li, Baofeng Chai

**Affiliations:** ^1^Institute of Loess Plateau, Shanxi University, Taiyuan, China; ^2^Shanxi Key Laboratory of Ecological Restoration on the Loess Plateau, Shanxi University, Taiyuan, China; ^3^Field Scientific Observation and Research Station of the Ministry of Education of Shanxi Subalpine Grassland Ecosystem, Shanxi University, Taiyuan, China; ^4^Department of Geography, Xinzhou Teachers University, Xinzhou, China

**Keywords:** bacterial community, shared taxa, unique taxa, diversity pattern, subalpine lakes

## Abstract

Bacterial communities have been described as early indicators of both regional and global climatic change and play a critical role in the global biogeochemical cycle. Exploring the mechanisms that determine the diversity patterns of bacterial communities and how they share different habitats along environmental gradients are, therefore, a central theme in microbial ecology research. We characterized the diversity patterns of bacterial communities in Pipahai Lake (PPH), Mayinghai Lake (MYH), and Gonghai Lake (GH), three subalpine natural lakes in Ningwu County, Shanxi, China, and analyzed the distribution of their shared and unique taxa (indicator species). Results showed that the species composition and structure of bacterial communities were significantly different among the three lakes. Both the structure of the entire bacterial community and the unique taxa were significantly influenced by the carbon content (TOC and IC) and space distance; however, the structure of the shared taxa was affected by conductivity (EC), pH, and salinity. The structure of the entire bacterial community and unique taxa were mainly affected by the same factors, suggesting that unique taxa may be important in maintaining the spatial distribution diversity of bacterial communities in subalpine natural freshwater lakes. Our results provide new insights into the diversity maintenance patterns of the bacterial communities in subalpine lakes, and suggest dispersal limitation on bacterial communities between adjacent lakes, even in a small local area. We revealed the importance of unique taxa in maintaining bacterial community structure, and our results are important in understanding how bacterial communities in subalpine lakes respond to environmental change in local habitats.

## Introduction

Aquatic bacteria are important components of lake ecosystems, having a high level of species diversity and playing essential roles in global biogeochemical cycles. Considerable evidence indicates that bacteria are essential to lake food web (Sanmukh et al., 2012; Cavicchioli, 2015), exert top-down control on other microbial communities and are symbiotic with lake organisms (such as planktonic algae, protozoa, fungi, and metazoa; Liu et al., 2015a, 2019; Xue et al., 2018). It is clear that bacteria are active and potentially significant players in water biological processes. This means that understanding the diversity of aquatic bacteria and their biogeographical patterns will help to explain the variations in ecosystem functioning, and ultimately to predict ecosystem responses to current and future environmental changes (Hanson et al., 2012). The majority of natural microbial communities is composed of a few abundant taxa and a large number of rare taxa (Liu et al., 2015b). However, abundant taxa are not necessarily the shared taxa of the habitat (Zhang et al., 2018), also the rare taxa are not necessarily the unique taxa of a certain habitat. Biogeographical patterns of shared and unique bacterial taxa in different habitats have been under-researched compared with the abundant and rare bacterial taxa in lakes and reservoirs (Baltar et al., 2015; Wu et al., 2016; Xue et al., 2018). Shared taxa are species that exist in every habitat within a certain boundary, which means that they have a stronger diffusion level and adaptability (Miura et al., 2019). Unique taxa are more restricted in habitat range, and their distribution is strongly affected by habitat conditions and distance between habitats (Mckenzie et al., 2012; Miura et al., 2019). It seems that both rare and unique taxa are mainly affected by environmental factors, but unique taxa have strict habitat specificity. Some studies have shown that abundant and rare bacterial taxa are distinctly different in diversity and biogeographical patterns, and rare bacterial taxa have a much smaller chance of successful diffusion than abundant taxa (Liu et al., 2015b; Jiao et al., 2017; Mo et al., 2018; Xue et al., 2018). However, little is known about the biogeographical basic patterning of shared and unique bacterial taxa, and to what extent lakes share bacterial taxa in a local area.

Most bacterial species are considered to be cosmopolitan because they have been found across biogeographic regions in multiple habitats, such as soil, sediment, lakes, and the sea (Hanson et al., 2012). In fact, bacteria are widely shared at phylum and class level, whereas at species or operational taxonomic unit (OTU) level they have habitat specificity. There is evidence of identical bacterial community composition in global oceans (Gibbons et al., 2013). With respect to community structure, however, regional endemism has been seen in bacteria, with some taxa reportedly being restricted to distinct geographical regions (Oakley et al., 2010; Kumar et al., 2017). Few studies have investigated the details of shared bacterial taxa community distribution in different habitats (Moitinho-Silva et al., 2014), or sought to understand the main drivers of bacterial diversity. Microbes display diverse biogeographical patterns, ranging from cosmopolitanism to provincialism (Hanson et al., 2012), but the underlying mechanisms that generate and maintain those patterns at a distinct range of spatial scales and habitats remain largely under explored (Meyer et al., 2013). Only a few studies have shown significant differences in the bacterial community structure of shared and unique taxa in the phyllosphere of forest, vineyard, and accompanying weed plants in local and regional areas (Samad et al., 2017; Miura et al., 2019). Those studies also showed the important influence of the environment on the structure and composition of a microbial community. Environmental factors, such as temperature (Callieri et al., 2016), dissolved oxygen (Di Cesare et al., 2015), and nutrient status (Liu et al., 2017), have powerful effects on the microbial community structure in alpine and subalpine lakes. Indeed, spatial processes are also important in producing and maintaining microbial diversity (Roguet et al., 2015). Little is known, however, about the mode of action and intensity of these regulatory mechanisms on different taxa.

The Ningwu subalpine natural lakes were formed in the Cenozoic Quaternary glacial period, approximately 3 million years ago (Wang et al., 2014). A group of 15 upland natural freshwater lakes of different sizes appeared on the planation surface; now, there are only three perennial lakes: Mayinghai, Pipahai, and Gonghai. Their water levels are declining yearly as a result of climate change and human disturbance (Liu et al., 2016). A study of Ningwu subalpine lakes (Zhang et al., 2012) found a clear difference in phytoplankton community structure, but the distribution of bacterial communities in Mayinghai, Pipahai, and Gonghai lakes, which were formed at the same time and in the same way is still unclear. The main purpose of our study was to explore bacterial community distribution, and its driving factors, in the three lakes. We characterized the microbiota, paying particular attention to three questions. (i) Are the structures of bacterial communities similar in the three lakes with the same formation conditions and similar climate? (ii) How do shared and unique taxa affect the assemblage of the entire bacterial community structure? and (iii) Can unique OTUs indicate habitat specificity? To achieve our aims, we used community DNA-based amplicon sequencing targeting the bacterial 16S rRNA gene region to conduct analyses.

## Materials and Methods

### Site Description

**Figure 1:**
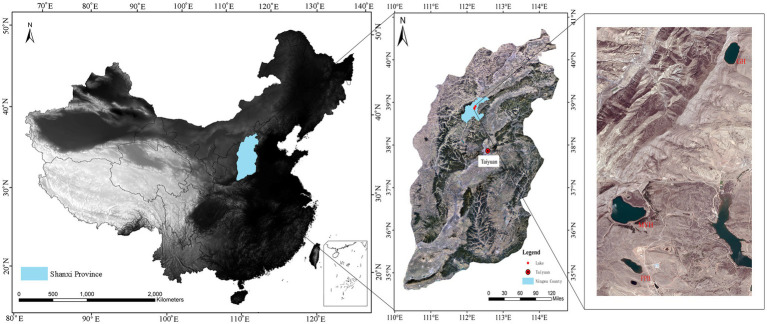
Map showing the location of sampling sites and spatial distribution of Pipahai Lake (PPH), Mayinghai Lake (MYH), and Gonghai Lake (GH) in the Ningwu County of Shanxi, China.

The study area was in Ningwu County, Shanxi Province, in the northern margin of the Chinese Loess Plateau, and three sampling sites were Pipahai Lake (PPH), Mayinghai Lake (MYH), and Gonghai Lake (GH; Figure 1). The size and area of these three lakes vary, and their altitude, maximum depth, and surface area appear in Table 1. The lakes are hydrologically closed basins and the main water source is precipitation. The research area has an East Asian monsoon climate with an annual mean temperature of 6.2°C. Annual precipitation is around 490 mm, of which more than 65% rainfalls during the summer (from June to August).

**Table 1:** Brief description of the sampling sites in the Ningwu subalpine lakes.

Parameter	PPH	MYH	GH
Location	38.85°N, 112.21°E	38.87°N, 112.20°E	38.91°N, 112.23°E
Elevation (m)	1776	1774	1854
Number of sampling points	3	4	5
Surface area (km^2^)	~0.21	~0.58	~0.36
Max depth (m)	~4.5	~6.4	~8.5

### Water Sampling

Water samples were collected every 2 m from top to bottom using a Plexiglass^®^ water sampler (LB-800, Qingdao, China) at the center of the three lakes in July 2017. Due to the different depth of the lake, there are 3, 4, and 5 sampling points in PPH, MYH, and GH, respectively (Table 1). Water was sampled three times at each sampling point and each repetition took 1 L water. In total, nine samples were collected from PPH, 12 samples from MYH, and 15 samples from GH. Approximately 2.5 L of the water sample from each sampling site was filtered in the laboratory using a sterile 0.2 μm pore size membrane filters (Millipore, Jinteng, Tianjin, China) for DNA extraction. Filters with retaining biomass were sealed and stored at −80°C until analysis. The remaining 0.5 L water was used for analysis of physicochemical properties.

### Physicochemical Measurements of Water Samples

For each sample, water physical parameters: temperature (T), pH, dissolved oxygen (DO), electric conductivity (EC), salinity (SAL), nitrate (${\mathrm{NO}}_{3}^{-}$), and ammonium (${\mathrm{NH}}_{4}^{+}$) content were measured *in situ* using a portable water multiparameter quality monitor (Aquaread AP-2000, England, United Kingdom). In the laboratory, we measured total nitrogen (TN), nitrite (${\mathrm{NO}}_{2}^{-}$), sulfate (${\mathrm{SO}}_{4}^{2-}$), and phosphate (${\mathrm{PO}}_{4}^{3-}$) content using an automated discrete analyzer (DeChem-Tech., CleverChem380, Hamburg, Germany); total carbon (TC), total organic carbon (TOC), and inorganic carbon (IC) content were measured using a TOC analyzer (Shimadzu, TOC-VCPH, Shimane, Japan).

### DNA Extractions, PCR Amplification, and High-Throughput Sequencing

Filters with retained biomass were cut into pieces and placed into centrifuge tubes for DNA extraction using the Fast soil DNA SPIN extraction kits (OMEGA Bio-tek Inc., Norcross, GA, United States) as described in the manufacturer’s protocol. We analyzed 12 samples (mixing the three repeats evenly for a sample from each sampling point) for bacterial communities. The primer pair 338F (5'-ACTCCTACGGGAGGCAGCA-3') and 806R (5'-GGACTACHVGGGTWTCTAAT-3') was used to amplify the V3-V4 hypervariable region of the 16S rDNA gene in bacteria. The PCR reactions were performed in triplicate using a 25 μl mixture containing 2.5 μl 10 × buffer (containing Mg^2+^), 2 μl of 2.5 mm dNTPs, 0.5 μl of each primer (10 μm), 0.25 μl of 5 U Fast Pfu polymerase, 1 μl of 10 ng template DNA, and 18.25 μl of ultrapure water. Thermal cycling consisted of initial denaturation at 98°C for 2 min followed by 26 cycles of denaturation at 98°C for 15 s, annealing at 55°C for 30 s, and an extension at 72°C for 30 s, with a final extension at 72°C for 5 min. Triplicates of PCR products were pooled and purified by the Agarose Gel DNA purification kit (TIANGEN, Tianjing, China) and quantified with the NanoDrop^™^ 8000 (Thermo Scientific, Massachusetts, United States) device. After purification and quantification of the PCR product, a genomic DNA library was constructed on an Illumina MiSeq platform in accordance with the manufacturer’s instruction manual (Majorbio Bio-Pharm Technology Co. Ltd., Shanghai, China).

### Nucleic Acid Sequences

The sequence data of bacterial 16S rDNA genes were submitted to the NCBI GenBank^1^ (accession number SRP131941).

### Bioinformatics Data Analysis

Before analysis, raw sequencing reads were demultiplexed and quality filtered using QIIME (version 1.9.1). The low-quality sequences were filtered using the following criteria: sequences that had a length of <150 bp, sequences that had average Phred scores of <20, sequences that contained ambiguous bases, and sequences that contained mononucleotide repeats of >8 bp. Paired-end reads were assembled using FLASH and Trimmomatic (Magoč and Salzberg, 2011). After chimera detection, the remaining high-quality sequences were clustered into OTUs at 97% sequence identity by UCLUST (Edgar, 2010). A representative sequence was selected from each OTU using default parameters. OTU taxonomic classification was conducted by BLAST search of the representative sequences set against the silva132 database using the best hit (Altschul et al., 1997). A OTU table was generated to record the abundance and taxonomy of every OTU in each sample. To minimize the differences in sequencing depth across samples, all were normalized to the number of sequences in the smallest data set for further analysis. Sequence data analyses were mainly performed using QIIME and R packages (version 3.3.1).

### Statistical Analysis

The alpha diversity of bacterial communities in each habitat was compared using observed OTU richness and the Shannon diversity index. Shapiro-Wilk tests were used to test the normality of physicochemical factors and alpha diversity data, and no violations of normality were detected. One-way ANOVA was used to assess the differentials in physicochemical factors and alpha diversity in the three habitats (PPH, MYH, and GH), and a least significant difference test was used for multiple comparisons by SPSS 20.0. The correlation between environmental factors and dominant sheared and unique taxa was expressed by a Spearman correlation coefficient. Beta diversity analysis was performed to investigate the structural variation in microbial communities across habitats using weighted UniFrac distance visualized with a hierarchical clustering tree, and Bray-Curtis distance metrics visualized *via* non-metric multidimensional scaling (NMDS). The differences in the three lake communities were examined using the ANOSIM statistics in the vegan package of R-3.3.1 (Oksanen et al., 2013). All environmental factors were selected by stepwise regression and the Monte Carlo permutation test; finally, environmental factors with the variance inflation factor (VIF) of less than 10 were retained for redundancy analysis (RDA; Supplementary Table S1). To prove whether appropriate habitat enabled the presence of unique taxa, we then determined which taxa were highly related to their habitat by identifying indicator species using the R package indicspecies (Oksanen et al., 2013). This analysis calculates an indicator value (IndVal) that measures the association between OTUs with each habitat and then identifies the taxa corresponding to the highest association value. We defined indicator OTUs based on an IndVal of >0.80 and a value of *p* < 0.05 assessed after 999 permutation tests (Miura et al., 2019). RDA was used to identify the correlation among the variables (environmental factors and space distance) and bacterial community composition. Firstly, before RDA analysis, six environmental factors with a VIF below 10 were preselected; then, the environmental variables and spatial distance were forward selected and only those with significant impact on community structure were retained for RDA (Supplementary Table S5). The contribution of environmental factors and space distance with the variations in the entire bacterial community and unique taxa was measured by variance partitioning analysis (VPA; McArdle and Anderson, 2001) in CANOCO 5.0. The spatial distance was expressed by the principal coordinates of neighboring matrices (PCNM). PCNM eigenfunctions were computed across the lake center locations, and computationally significant variables (*p* < 0.05) were selected in the R vegan package. The confidence interval of all statistical analyses was 95% (*p* < 0.05).

## Results

### Physicochemical Properties

The concentrations of TN, ${\mathrm{NO}}_{3}^{-}$, ${\mathrm{NH}}_{4}^{+}$, TC, IC, TOC, ${\mathrm{SO}}_{4}^{2-}$, pH, and EC were significantly different in the three lakes (Table 2). The concentration of TOC was higher in PPH than in GH and MYH; however, the other eight factors were highest in GH (*p* < 0.05). The relatively higher concentration of organic carbon and a lower pH in the PPH water samples indicated that the lake was more polluted and showed a trend of acidification compared with the other two lakes (Table 2).

**Table 2:** Water physicochemical characteristics of studied lakes.

Parameter	PPH	MYH	GH
T (°C)	23.69 ± 0.32a	22.89 ± 0.35ab	21.40 ± 0.50b
pH	7.58 ± 0.02c	7.98 ± 0.14b	8.51 ± 0.04a
DO (mg/L)	7.92 ± 0.57b	10.64 ± 0.79a	9.24 ± 0.23ab
EC (uS/cm)	476.33 ± 1.54b	409.75 ± 2.11c	935.67 ± 3.28a
SAL(ng/L)	6.25 ± 0.39a	7.91 ± 0.64a	7.49 ± 0.37a
TN (mg/L)	1.79 ± 0.12b	1.01 ± 0.04c	2.71 ± 0.09a
${\mathrm{NO}}_{3}^{-}$ (mg/L)	0.23 ± 0.01b	0.17 ± 0.02c	0.28 ± 0.01a
${\mathrm{NO}}_{2}^{-}$ (mg/L)	0.01 ± 0.00b	0.01 ± 0.00b	0.03 ± 0.01a
${\mathrm{NH}}_{4}^{+}$ (mg/L)	1.44 ± 0.10b	0.73 ± 0.04c	2.09 ± 0.09a
TC (mg/L)	79.72 ± 0.81b	60.42 ± 0.25c	131.31 ± 0.83a
IC (mg/L)	51.87 ± 0.40b	45.79 ± 0.25c	110.39 ± 0.88a
TOC (mg/L)	27.86 ± 0.48a	14.64 ± 0.27c	20.91 ± 0.20b
C/N	46.21 ± 3.46b	59.82 ± 2.45a	49.23 ± 1.88b
${\mathrm{SO}}_{4}^{2-}$ (mg/L)	52.68 ± 0.52b	48.82 ± 1.03c	59.48 ± 0.41a
${\mathrm{PO}}_{4}^{3-}$ (mg/L)	0.25 ± 0.05a	0.31 ± 0.06a	0.40 ± 0.07a

The data were shown as the means ± standard error. T represents temperature; DO represents dissolved oxygen; EC represents electro conductibility; SAL represents salinity; TN represents total nitrogen; $NO_{3}^{-}$ represents nitrate; $NO_{2}^{-}$ represents nitrite; $NH_{4}^{+}$ represents ammonium; TC represents total carbon; IC represents inorganic carbon; TOC represents organic carbon; C/N represents ratio of total carbon to total nitrogen; $SO_{4}^{2-}$ represents sulfate; and $PO_{4}^{3-}$ represents phosphate. Significant differences between samples were determined using one-way ANOVA at *p* < 0.05 and the different letters indicate significant differences.

### Community Composition of Lake Water Bacteria

**Figure 2:**
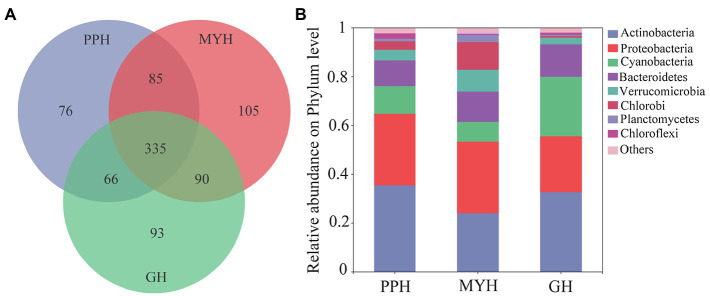
Bacterial community compositions in water samples in PPH, MYH, and GH. **(A)** Venn diagram showing the operational taxonomic units (OTUs) number in the three lakes and **(B)** the composition of the dominant bacterial phyla (with average relative abundance >1%) across the three lakes, where sequences that have a mean relative abundance <1% were assigned to others.

We obtained 375,171 high-quality bacterial 16S rDNA gene sequences and 850 OTUs by high-throughput sequencing of all water samples. The sample from MYH had higher OTU richness (615 OTUs), based on the number of OTUs, whereas samples from PPH and GH displayed considerably lower richness, with 562 OTUs and 584 OTUs, respectively (Figure 2A). The composition of bacterial communities differed in all three lakes, and although the eight dominant phyla were shared, their relative abundance varied among the three lakes (Figure 2B). The eight dominant phyla were *Actinobacteria* (relative abundance 24.46–36.18%), *Proteobacteria* (22.78–29.12%), *Cyanobacteria* (8.41–23.85%), *Bacteroidetes* (10.43–13.33%), *Verrucomicrobia* (2.60–8.70%), *Chlorobi* (0.72–11.11%), *Planctomycetes* (0.49–2.88%), and *Chloroflexi* (0.51–2.15%; Figure 2B).

**Figure 3:**
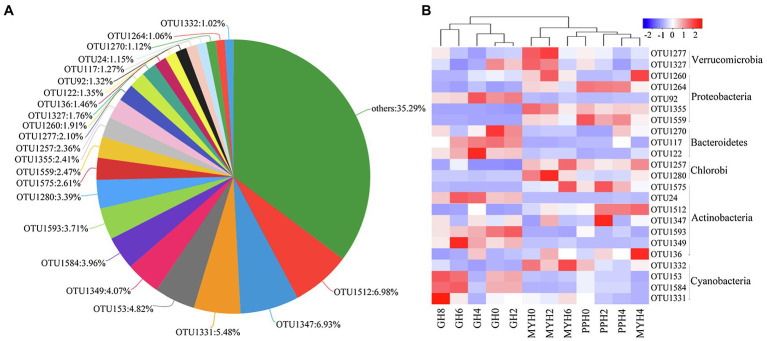
Shared OTUs in the three lakes. **(A)** Pie chart showing the dominant shared OTUs (with average relative abundance >1% and OTUs with relative abundance less than 1% were merged into others). **(B)** Heat map showing the distribution pattern of dominant shared OTUs in 12 water samples.

The three lakes shared 335 OTUs (Figure 2A), including 23 dominant OTUs (with relative abundance greater than 1%; Figure 3A). The OTUs shared by the three lakes contained 86.08% of the total bacterial abundance in PPH. In MYH and GH, the shared OTUs accounted for 90.33 and 81.49% of the total OTUs, respectively. The shared dominant OTUs belonged to six different bacterial phyla and their abundance varied with sampling site (Figure 3B). Of these 23 OTUs, seven belonged to *Actinobacteria*, five to *Proteobacteria*, four to *Cyanobacteria*, three to *Bacteroidetes*, two to *Verrucomicrobia*, and two to *Chlorobi* (Figure 3B). A significant correlation was found between the change in abundance of the dominant shared OTUs and environmental parameters (Supplementary Table S2). The changes in EC, carbon (TC and IC), and nitrogen (TN, ${\mathrm{NO}}_{3}^{-}$, and ${\mathrm{NH}}_{4}^{+}$) concentrations in the 15 physicochemical factors tested had significant correlations with the abundance of dominant shared OTUs (Supplementary Table S2).

The number of unique bacterial taxa in each lake differed and was 76 (PPH), 105 (MYH), and 93 (GH; Figure 2). After screening by IndVal (IndVal > 0.8), we found 54 unique OTUs in PPH, 15 unique OTUs in MYH, and 21 unique OTUs in GH (Supplementary Table S3). The most abundantly unique taxa in PPH were OTU1556, belonging to genus *norank_f__0319-6G20* family *LiUU-11-161* order *Sphingobacteriales* class *Sphingobacteriia* phylum *Bacteroidetes*; in MYH was OTU1315, belonging to genus *Methyloparacoccus* family *Methylococcaceae* order *Methylococcales* class *Gammaproteobacteria* phylum *Proteobacteria*; and, in GH was OTU17 belonging to genus *MWH-UniP1_aquatic_group* family *Alcaligenaceae* order *Burkholderiales* class *Betaproteobacteria* phylum *Proteobacteria*. The greatest diversity of unique taxa in all three lakes belonged to *Proteobacteria*, and the numbers of these OTUs in PPH, MYH, and GH were 28, 7, and 7, respectively (Supplementary Table S3). A significant correlation was found between the abundance of the top five unique OTUs and EC, as well as between carbon (TC and IC) and nitrogen (TN, ${\mathrm{NO}}_{3}^{-}$, and ${\mathrm{NH}}_{4}^{+}$; Supplementary Table S4).

### Richness and Diversity of the Bacterial Community

**Figure 4:**
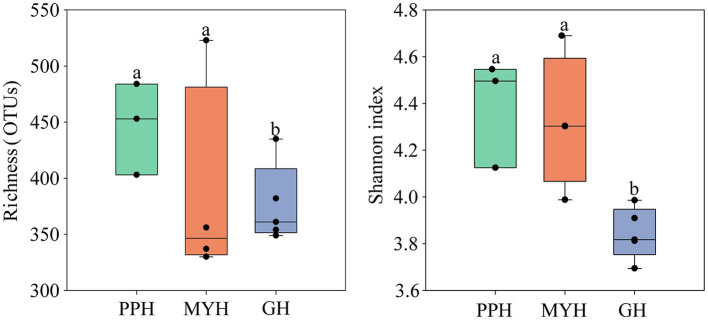
Variation in alpha diversity of bacterial communities on the PPH, MYH, and GH. The error bars represent standard deviations of means. Different letters indicate a significant difference between the three lakes according to LDS multiple comparisons (*p* < 0.05).

Alpha-diversity characteristics were estimated using the observed OTUs and Shannon’s index. One-way ANOVA showed a significant difference in OTU number (*F* = 5.55, *p* < 0.05) and Shannon diversity in the three lakes (*F* = 8.46, *p* < 0.01; Figure 4). GH had the lowest mean values of OTUs (366.20 ± 35.20) and Shannon diversity (3.84 ± 0.11) among these three lakes, and multiple comparisons showed that differences in alpha-diversity indexes between GH and the other two lakes were statistically significant (Figure 4). A significant correlation was found between environmental factors and alpha diversity of the bacterial community. The trend of bacterial community richness had a significant correlation with pH, DO, SAL, ${\mathrm{SO}}_{4}^{2-}$, and ${\mathrm{PO}}_{4}^{3-}$, and the change in the Shannon index was significantly correlated with pH, EC, TN, ${\mathrm{NO}}_{3}^{-}$, ${\mathrm{NH}}_{4}^{+}$, IC, and TOC (Table 3). The results showed that the environmental gradient among the lakes could lead to a change in the alpha diversity of a bacterial community.

**Table 3:** The Spearman correlations between environmental factors and alpha diversity of bacterial communities.

Parameters	OTUs	Shannon
T (°C)	−0.308	0.315
pH	−0.725^**^	−0.785^**^
DO (mg/L)	−0.916^**^	−0.490
EC (uS/cm)	0.266	−0.636^*^
SAL (ng/L)	−0.909^**^	−0.371
TN (mg/L)	0.182	−0.720^**^
${\mathrm{NO}}_{3}^{-}$ (mg/L)	0.147	−0.615^*^
${\mathrm{NO}}_{2}^{-}$ (mg/L)	0.296	−0.176
${\mathrm{NH}}_{4}^{+}$ (mg/L)	0.21	−0.692^*^
TC (mg/L)	−0.497	0.133
IC (mg/L)	−0.077	−0.811^**^
TOC (mg/L)	0.077	−0.713^**^
C/N	0.378	−0.168
${\mathrm{SO}}_{4}^{2-}$ (mg/L)	0.622^*^	0.189
${\mathrm{PO}}_{4}^{3-}$ (mg/L)	−0.622^*^	−0.406
Depth	0.383	−0.147
Area	−0.484	0.048

OTUs represent the number of operational taxonomic units in the bacterial community obtained by high-throughput sequencing, and the values in the table represent the correlation coefficient (*r*).

** *p* < 0.01;

* *p* < 0.05.

### Spatial Distribution of Bacterial Community and Driving Factors

**Figure 5:**
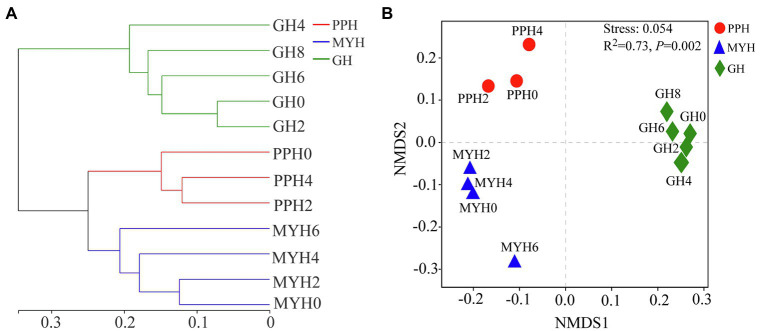
Bacterial community structure analyses shown as hierarchical clustering tree **(A)** and non-metric multidimensional scaling plots **(B)** based on Bray-Curtis distance (in OTU level) for pairwise differences between datasets originating from the PPH, MYH, and GH.

A hierarchical clustering tree based on weighted UniFrac distance showed that the spatial distribution of the bacterial community in GH was different from that in MYH and PPH (Figure 5A). The NMDS based on Bray–Curtis distance showed that the spatial distribution of the bacterial community differed in the three lakes (Figure 5B), and this was further confirmed by PERMANOVA analysis (ANOSIM, *R*^2^ = 0.73, *p* = 0.002). The structure of shared taxa (ANOSIM, *R*^2^ = 0.727, *p* = 0.006) and unique taxa (ANOSIM, *R*^2^ = 0.772, *p* = 0.001) also created significant differences among the three lakes (Supplementary Figure S1).

**Figure 6:**
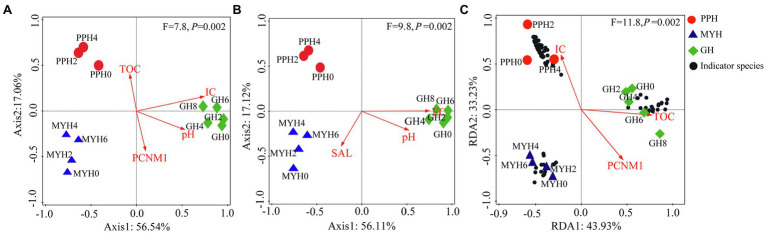
Redundancy analyses of environmental factors and spatial distance on bacterial community structure. **(A)** Entire bacterial community, **(B)** shared taxa, and **(C)** unique taxa.

RDA analysis showed that the distribution pattern of the entire bacterial community was mainly affected by TOC, IC, pH, and PCNM1 (*F* = 7.8, *p* = 0.002; Figure 6A), and the distribution pattern of shared taxa was mainly affected by EC, pH, and SAL (*F* = 9.8, *p* = 0.002; Figure 6B), whereas unique taxa structure was mainly affected by TOC, IC, and PCNM1 (*F* = 11.8, *p* = 0.01; Figure 6C).

**Figure 7:**
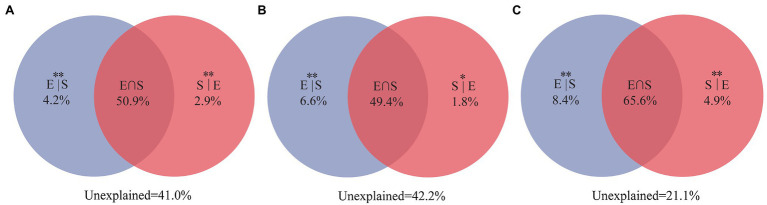
Variation partitioning analyses showing the percentages of variance in water bacterial communities explained by environmental factors and spatial distance. **(A)** For the entire community, including three environmental factors (TOC, IC, and pH) and spatial distance; **(B)** for the entire community, including two selected environmental factors (TOC and IC) and spatial distance; and **(C)** for unique taxa, including two selected environmental factors (TOC and IC) and spatial distance. The variation explained by pure spatial and environmental factors correspond to the bacterial community without the effect of the other by the ANOVA permutation tests. ^*^p < 0.05 and ^**^p < 0.01. S|E, pure spatial variation; E|S, pure environmental variation; S∩E share explained variation; and 1 − S|E − E|S − S∩E = unexplained variation.

Variance partitioning analysis revealed that 59% of the variation for the entire community were significantly explained by the selected three environment variables (TOC, IC, and pH) and spatial distance. Among them, environmental variables and spatial distance independently explained 4.2 and 2.9%, respectively (Figure 7A). At the same time, the entire community was significantly explained 57.8% of the selected two environment variables (TOC and IC) and spatial variables. Among them, environmental variables and spatial distance independently explained 6.6 and 1.8%, respectively (Figure 7B). For unique taxa, the combination of environmental variables and spatial distance explained 78.9% of the observed variation (Figure 7C), while environmental variables and spatial distance explained 8.4 and 4.9%, respectively. VPA showed that environmental variables and spatial distance explained more than 50% of the composition of the entire community. Notably, pH only explained 1.2% proportion in the composition of the entire community.

## Discussion

### Environmental Variability of Bacterial Community Composition

To explore the structure and function of an ecosystem, it is necessary to understand the number and kinds of microbial taxa within a community (Shafi et al., 2017). In agreement with the previous studies on freshwater lakes (Llirós et al., 2014), we found typical freshwater bacterial communities in the three lakes, with dominant microbiomes mainly comprising *Actinobacteria*, *Proteobacteria*, *Cyanobacteria*, *Bacteroidetes*, *Verrucomicrobia*, *Chlorobi*, *Chloroflexi*, and *Planctomycetes* groups (Figure 2B). The relative abundance of the predominant bacterial phyla differed among the lakes, and *Actinobacteria* and *Cyanobacteria*, in particular, varied significantly (Figure 2B). Several ecological factors affected the abundance of *Actinobacteria* and *Cyanobacteria*. Important factors influencing *Actinobacteria* included pH, organic carbon content (Llirós et al., 2014), and water temperature (Tang et al., 2017); *Cyanobacteria* was mainly influenced by water temperature, pH, and trophic status (especially nitrogen and phosphorus content; Liao et al., 2016). It was not difficult to understand why the abundance of *Actinobacteria* and *Cyanobacteria* was the lowest in MYH, because this lake had the lowest nutrition levels (Table 2).

Shared OTUs number accounts for more than half of the total number in each lake (Figure 2). This phenomenon is common, whether in a soil bacterial community at a regional scale (Pereira et al., 2012) or in a water bacterial community at a local scale (Yu et al., 2018). This kind of pattern formation is mainly because the shared OTUs have a wide range of habitats. Some research has shown that pan-habitat species have a wide habitat tolerance, good exploitation ability, and high functional plasticity (Székely and Silke, 2014), indicating that they have strong adaptability, and that a moderate environmental gradient would not have a significant impact on their diversity. We found that the abundance of the dominant shared OTUs was different among the three lakes (Figure 3B), and this was significantly related to EC, carbon (TC and IC), and nitrogen (TN, ${\mathrm{NO}}_{3}^{-}$, and ${\mathrm{NH}}_{4}^{+}$; Supplementary Table S2). Water nutritional status (especially carbon and nitrogen) has been shown to significantly impact bacterial composition (Tang et al., 2017). The 23 dominant shared OTUs belonged to six dominant phyla. Of these, most species were from *Actinobacteria*; and OTU1512, which had the highest relative abundance (6.98%), also belonged to *Actinobacteria* (Figure 3). This result was similar to the previous observations in a freshwater lake, where the bacterial community from phylum to genus was mainly composed of *Actinobacteria* (Tang et al., 2017). A possible explanation is that *Actinobacteria* have different growth and division patterns, and their habitat niche is broad (Székely and Silke, 2014). This may explain their cosmopolitan behavior.

Most unique taxa were in PPH and the least in MYH (Supplementary Table S3). We found that the unique dominant species in the three lakes belonged to different bacterial genera, and the diversity of unique species was the highest in *Proteobacteria* (Supplementary Table S3). These results showed that the heterogeneity of habitat determines the functional differences in these groups. However, they also showed that *Proteobacteria* play an important role in the stability of the community structure and the maintenance of functional diversity (Zhou et al., 2020). The most abundant unique taxa in PPH (OTU1556) belonged to genus *norank_f_LiUU-11-161*, which can decompose ammonia nitrogen in water (Sun et al., 2019). The most abundant unique taxa in MYH (OTU1315) belonged to *Methyloparacoccus*. This is a heterotrophic microorganism that obtains energy by decomposing methyl compounds (Li et al., 2020). The most abundant unique taxa in GH (OTU17) belonged to the genus *MWH-UniP1*_aquatic_group. Members of this genus commonly live in alkaline water with high nutrient concentrations (Krett et al., 2017), obtaining energy through denitrification. Unique taxa exist only in a specific habitat and just like habitat specialists, which were preserved by environmental filtration, and that their existence would largely depend on these specific or combined with environmental factors (Székely and Silke, 2014). Each lake had its own unique bacterial taxa, and there was a certain correlation with carbon, nitrogen, and electrical conductivity (Supplementary Table S4). This result can answer our third question, showing that the unique taxa cannot represent the specificity of their habitat.

### Factors Shaping Bacterial Community Diversity and Structure in Subalpine Freshwater Lakes

Compared with PPH and MYH, GH exhibited higher nutrient loading (Table 2), but we observed a lower alpha diversity in the bacterial community (Figure 4). Contrary observations were reported by Zhao (Zhao et al., 2017) who suggested that a higher nutrient state could weaken niche selection by reducing competition for resources and providing more diverse resources to some microbial species, which may contribute to higher diversity. Of course, our results were not difficult to understand, because the GL is the deepest of the three lakes (Table 1), and there is an obvious vertical gradient of nutrients (Wang et al., 2019), which leads to the uneven distribution of bacterial community.

Several studies at different spatial scales have shown that ecological factors are responsible for shaping bacterial community structure, such as physicochemical characteristics (Yang et al., 2016), eukaryotic plankton community composition (Yang et al., 2016), and spatial distance (Zinger et al., 2014). Few studies have investigated the effects of these factors on the composition and structure of shared and unique bacterial taxa. Our results show significant differences in the spatial distribution of the entire bacterial community (*p* = 0.002; Figure 5), shared taxa (*p* = 0.006), and unique taxa (*p* = 0.001) among the three lakes (Supplementary Figure S1; Figure 6). VPA analysis revealed that the dissimilarity in the entire bacterial community structure among the three lakes was related to environmental factors and spatial distance. The influence of environmental factors was greater than space distance, and TOC and IC were the main environmental factors (Figure 7A). The results also showed that the spatial distribution pattern of the bacterial community in subalpine lakes was affected by dispersal limitation even at a small spatial range (approximately 10 km; Figure 1). Although study found that the similarity of bacterial communities is inversely proportional to the geographical distance at small spatial scales in marine waters (Mo et al., 2018), the role of diffusion limitation is higher in subalpine lakes with clear boundaries than in the indeterminate boundaries water. Nutrient concentrations (especially carbon content) often influence biomass and the taxonomic composition of a heterotrophic microbial community in water (Fujii et al., 2012). The assimilation of organic carbon by heterotrophic microorganisms is a key step in the carbon flow in marine and freshwater ecosystems. Our results also confirmed that the differences in TOC and IC content were key factors controlling bacterial diversity and community composition in freshwater environments (Figure 6). Most mountain lakes are characterized by low nutrient concentrations (Fujii et al., 2012), as were our study areas. Our results showed that the area of a lake is inversely proportional to the concentration of organic carbon (Tables 1 and 2). Therefore, organic carbon input from terrestrial sources can be considered as an important carbon supply for microbial communities in such lakes. Several studies have confirmed that the pH gradient has a significant effect on the structure of a microbial community (Fujii et al., 2012), but pH had a small rate of explanation (only 1.2%) on the distribution of a bacterial community in this study (Figures 7A,B). Compared with another study (Lindstrom et al., 2005), we found a narrow range of pH (7.58–8.51) in our study, and this may have been the main reason for the less significant impact on bacterial community structure. The spatial distribution pattern of the entire bacterial community and unique taxa was mainly affected by the same factors (Figure 7), suggesting that unique taxa may play an important role in maintaining the spatial distribution diversity of a bacterial community in subalpine natural freshwater lakes. Species traits of unique taxa, whose successful spread is difficult owing to their small numbers, determined the difference in spatial distribution. Besides, species traits affect the interaction patterns and the organization of microbial dispersal interaction networks (Hanson et al., 2012). In this study, the effect of dispersal limitation on the entire bacterial community was mainly reflected in the effect of dispersal limitation on unique taxa (Figure 6). The change of composition and distribution of unique taxa more likely to depend on habitat properties (Pandit et al., 2009), which leads to a high extinction risk of them, and then affects the diversity and function of the entire bacterial community. Unique taxa are known to have a limited niche, but the highest fitness in their optimal habitat. At the same time, these groups occupy an important position in the ecological network, indicating their importance in the food web (Monard et al., 2016). In view of the fact that ecosystem functions may be influenced by the characteristics and relative abundance of unique taxa, an understanding of the local distribution of unique taxa is needed to improve the prediction of ecosystem response to global change.

## Conclusion

In this study, we found that bacterial community composition, diversity, and structure changed markedly among the three subalpine lakes. Variations in bacterial community composition and alpha diversity strongly correlated with the physical and chemical parameters of lake water. Moreover, although most OTUs were shared by the lakes, there were some unique OTUs in every lake. The composition of the unique OTUs was regulated by the nutritional status (especially carbon sources) of their habitat, suggesting that the type and amount of carbon sources in subalpine oligotrophic lakes determine the number and distribution of unique OTUs. The spatial distribution patterns of bacterial communities were mainly driven by carbon content and spatial distance, highlighting that the differences in bacterial communities structure strongly depended on the fluctuation in nutritional status caused by surrounding land use types around the lakes. At the same time, diffusion restriction also had an important effect on bacterial community structure in the closed small lakes. The spatial distribution pattern of the entire bacterial community and of unique bacterial taxa was mainly affected by the same factors. Finally, our results emphasized the importance of unique bacterial taxa in maintaining the diversity of entire bacterial communities in subalpine freshwater lakes.

## Data Availability Statement

The sequence data of bacterial 16S rDNA genes were submitted to the NCBI GenBank (accession number SRP131941), and the physical and chemical data of environment is not uploaded to the database.

## Author Contributions

BC and JL designed the study, performed the statistical analyses, and prepared the draft of the manuscript. JL, JS, MZ, ZL, and XL collected the water samples. BC, JL, and XL performed the experiments. JL advised on the figures and tables. All authors revised the manuscript and approved the final version.

### Conflict of Interest

The authors declare that the research was conducted in the absence of any commercial or financial relationships that could be construed as a potential conflict of interest.

## Footnotes

^1^https://www.ncbi.nlm.nih.gov/sra/?term=SRP131941

## Supplementary Material

The Supplementary Material for this article can be found online at: https://www.frontiersin.org/articles/10.3389/fmicb.2021. 669131/full#supplementary-material
