# Supplementary material for: Bacterial Community Spacing Is Mainly Shaped by Unique Species in the Subalpine Natural Lakes of China
Source: Front Microbiol. 2021 Jul 1;12:669131. doi: 10.3389/fmicb.2021.669131 (PMC8282455; doi:10.3389/fmicb.2021.669131)
Supplement: Supplementary file 1 [file Data_Sheet_1.doc]

**Bacterial Community Spacing is Mainly Shaped by Unique Species in the Subalpine Natural Lakes of China**

***Jinxian Liu***^1^, ***Jiahe Su***^1^, ***Meiting Zhang***^1^, ***Zhengming Luo***^1,2^, ***Xiaoqi Li***^1^, ***Baofeng Chai***^1^***

^1^Institute of Loess Plateau, Shanxi University; Shanxi Key Laboratory of Ecological Restoration on the Loess Plateau, Field Scientific Observation and Research Station of the Ministry of Education of Shanxi Subalpine Grassland Ecosystem, Taiyuan, China

^2^Department of Geography, Xinzhou Teachers University, Xinzhou, China

TABLE S1 The multicollinearity test results of 18 different factors

| Factors | VIF values | |
| --- | --- | --- |
|  | Before screening | After screening |
| T (℃) | 326.13 | 16.68 |
| pH | 168.90 | **9.67** |
| DO (mg/L) | 108.19 | 39.66 |
| EC (uS/cm) | 4671.32 | **3.51** |
| SAL(ng/L) | 65.74 | **6.99** |
| TN (mg/L) | 2048.43 | **9.20** |
| NO_3_^-^ (mg/L) | 2261.31 | 42.20 |
| NO_2_^-^ (mg/L) | 324.94 | 13.29 |
| NH_4_^+^ (mg/L) | 733.76 | 352.34 |
| TC (mg/L) | 430718.65 | 35998.96 |
| IC (mg/L) | 35933.36 | **9.21** |
| TOC (mg/L) | 3379.59 | **7.79** |
| C/N | 4671.32 | 482.88 |
| SO_4_^2-^ (mg/L) | 17880.27 | 225.99 |
| PO_4_^3-^ (mg/L) | 559.63 | 14.79 |
| PCNM1 | 1378.74 | **9.35** |
| Depth | 2111.75 | 32.52 |
| Area | 29524.41 | 1078.26 |

Note: variance inflation factors (VIF) less than 10 were considered to have no multicollinearity

**TABLE S2** Spearman correlation between dominant shared OTUs and environmental parameters

| Factors | T | pH | DO | EC | SAL | TN | NO_3_^-^ | NO_2_^-^ | NH_4_^+^ | C/N | TC | IC | TOC | SO_4_^2-^ | PO_4_^3-^ |
| --- | --- | --- | --- | --- | --- | --- | --- | --- | --- | --- | --- | --- | --- | --- | --- |
| OTU1277 | 0.42 | -0.25 | 0.20 | -0.56 | -0.01 | -0.58* | -0.57 | -0.54 | -0.57 | 0.22 | -0.55 | -0.32 | -0.69* | -0.40 | -0.01 |
| OTU1327 | 0.67* | 0.41 | 0.60* | -0.40 | 0.44 | -0.37 | -0.17 | -0.16 | -0.39 | 0.36 | -0.19 | -0.34 | -0.32 | -0.41 | 0.27 |
| OTU1260 | 0.30 | 0.09 | 0.36 | -0.76** | 0.62* | -0.73** | -0.57 | -0.18 | -0.75** | 0.65* | -0.67* | -0.81** | -0.64* | -0.54 | 0.12 |
| OTU1264 | 0.64* | -0.61* | -0.04 | -0.62* | -0.20 | -0.65* | -0.65* | -0.51 | -0.60* | 0.01 | -0.53 | 0.26 | -0.59* | 0.32 | -0.04 |
| OTU1355 | 0.55 | -0.47 | 0.09 | -0.80** | 0.08 | -0.81** | -0.90** | -0.70* | -0.77** | 0.29 | -0.74** | -0.08 | -0.85** | -0.21 | -0.04 |
| OTU1559 | 0.50 | -0.72** | -0.25 | -0.56 | -0.36 | -0.57 | -0.68* | -0.59* | -0.50 | -0.11 | -0.59* | 0.33 | -0.66* | 0.25 | -0.27 |
| OTU92 | -0.61* | 0.48 | -0.11 | 0.79** | 0.01 | 0.74** | 0.83** | 0.76** | 0.69* | -0.14 | 0.72** | 0.03 | 0.84** | 0.13 | 0.21 |
| OTU1331 | -0.29 | -0.06 | -0.24 | 0.36 | -0.37 | 0.25 | 0.18 | 0.28 | 0.21 | -0.11 | 0.21 | -0.11 | 0.22 | 0.02 | -0.35 |
| OTU1332 | 0.39 | -0.60* | -0.14 | -0.76** | -0.22 | -0.76** | -0.80** | -0.42 | -0.73** | 0.14 | -0.85** | -0.39 | -0.85** | -0.18 | -0.37 |
| OTU153 | -0.28 | 0.16 | -0.29 | 0.76** | -0.41 | 0.69* | 0.66* | 0.41 | 0.66* | -0.39 | 0.66* | 0.46 | 0.64* | 0.36 | -0.25 |
| OTU1584 | -0.32 | 0.27 | -0.21 | 0.88** | -0.38 | 0.87** | 0.80** | 0.49 | 0.84** | -0.52 | 0.82** | 0.57 | 0.82** | 0.45 | -0.16 |
| OTU1257 | 0.18 | -0.75** | -0.34 | -0.72** | -0.25 | -0.76** | -0.87** | -0.44 | -0.72** | 0.14 | -0.89** | -0.35 | -0.83** | -0.09 | -0.39 |
| OTU1280 | 0.36 | -0.54 | 0.04 | -0.85** | 0.08 | -0.87** | -0.94** | -0.63* | -0.85** | 0.38 | -0.85** | -0.41 | -0.90** | -0.27 | -0.30 |
| OTU117 | -0.36 | 0.50 | -0.11 | 0.82** | -0.05 | 0.82** | 0.93** | 0.74** | 0.80** | -0.30 | 0.82** | 0.42 | 0.91** | 0.38 | 0.09 |
| OTU122 | -0.64* | 0.66* | 0.20 | 0.60* | 0.35 | 0.60* | 0.58* | 0.47 | 0.55 | 0.08 | 0.59* | -0.26 | 0.64* | -0.30 | 0.29 |
| OTU1270 | -0.41 | 0.43 | -0.08 | 0.44 | 0.28 | 0.33 | 0.60* | 0.46 | 0.27 | 0.32 | 0.36 | -0.14 | 0.39 | -0.17 | 0.27 |
| OTU1347 | 0.02 | 0.15 | 0.34 | 0.06 | 0.05 | 0.22 | 0.27 | -0.23 | 0.25 | -0.27 | 0.16 | 0.14 | 0.08 | -0.06 | 0.38 |
| OTU1349 | -0.57 | 0.70* | 0.29 | 0.59* | 0.35 | 0.59* | 0.56 | 0.48 | 0.54 | 0.09 | 0.66* | -0.23 | 0.68* | -0.29 | 0.21 |
| OTU136 | 0.27 | -0.33 | 0.17 | -0.68* | 0.25 | -0.59* | -0.55 | -0.44 | -0.56 | 0.21 | -0.59* | -0.19 | -0.58* | -0.06 | 0.15 |
| OTU1512 | 0.38 | -0.54 | -0.03 | -0.64* | -0.01 | -0.57 | -0.57 | -0.51 | -0.52 | 0.01 | -0.58* | 0.08 | -0.57 | 0.23 | 0.01 |
| OTU1575 | 0.22 | -0.90** | -0.41 | -0.53 | -0.50 | -0.55 | -0.59* | -0.45 | -0.49 | -0.15 | -0.63* | 0.15 | -0.59* | 0.36 | -0.39 |
| OTU1593 | -0.15 | 0.68* | 0.18 | 0.80** | 0.11 | 0.80** | 0.88** | 0.61* | 0.75** | -0.21 | 0.91** | 0.42 | 0.88** | 0.27 | 0.25 |
| OTU24 | -0.45 | 0.49 | -0.10 | 0.91** | -0.08 | 0.90** | 0.84** | 0.60* | 0.86** | -0.36 | 0.90** | 0.46 | 0.94** | 0.33 | 0.14 |

Note: The unit of T is ℃, the unit of EC is uS/cm, the unit of SAL is ng/L, pH and C/N have not unit, The unit of other parameters all is mg/L. ***P* < 0.01; **P* < 0.05.

**TABLE S3** Composition and abundance of indicator species in the Pipahai lake (PPH), Mayinghai lake (MYH) and Gonghai lake (GH)

|  | OTU | Phylum | Class | Order | Family | Abundance |
| --- | --- | --- | --- | --- | --- | --- |
| PPH | OTU1605 | Acidobacteria | Acidobacteria | Blastocatellales | Blastocatellaceae__Subgroup_4_ | 20 |
|  | OTU1510 | Actinobacteria | Actinobacteria | Solirubrobacterales | unclassified_o__Solirubrobacterales | 19 |
|  | OTU1613 | Bacteroidetes | Cytophagia | Cytophagales | Cytophagaceae | 13 |
|  | OTU1509 | Bacteroidetes | Cytophagia | Cytophagales | Cytophagaceae | 18 |
|  | OTU1539 | Bacteroidetes | Cytophagia | Cytophagales | Cytophagaceae | 46 |
|  | OTU1551 | Bacteroidetes | Sphingobacteriia | Sphingobacteriales | Chitinophagaceae | 20 |
|  | OTU1552 | Bacteroidetes | Sphingobacteriia | Sphingobacteriales | Chitinophagaceae | 23 |
|  | OTU1623 | Bacteroidetes | Sphingobacteriia | Sphingobacteriales | env.OPS_17 | 72 |
|  | **OTU1556** | **Bacteroidetes** | **Sphingobacteriia** | **Sphingobacteriales** | **LiUU-11-161** | **130** |
|  | OTU1503 | Bacteroidetes | Sphingobacteriia | Sphingobacteriales | NS11-12_marine_group | 21 |
|  | OTU1513 | Bacteroidetes | Sphingobacteriia | Sphingobacteriales | PHOS-HE51 | 6 |
|  | OTU1681 | Bacteroidetes | Sphingobacteriia | Sphingobacteriales | unclassified_o__Sphingobacteriales | 11 |
|  | OTU1637 | Bacteroidetes | Sphingobacteriia | Sphingobacteriales | unclassified_o__Sphingobacteriales | 40 |
|  | OTU1616 | Bacteroidetes | unclassified_p__Bacteroidetes | unclassified_p__Bacteroidetes | unclassified_p__Bacteroidetes | 22 |
|  | OTU1524 | Chloroflexi | Chloroflexia | Chloroflexales | Chloroflexaceae | 22 |
|  | OTU1581 | Cyanobacteria | Cyanobacteria | norank_c__Cyanobacteria | norank_c__Cyanobacteria | 6 |
|  | OTU1557 | Cyanobacteria | Cyanobacteria | SubsectionI | FamilyI_o__SubsectionI | 7 |
|  | OTU1487 | Elusimicrobia | Elusimicrobia | Lineage_I | Unknown_Family_o__Lineage_I | 2 |
|  | OTU1646 | Proteobacteria | Alphaproteobacteria | Rhizobiales | unclassified_o__Rhizobiales | 8 |
|  | OTU1629 | Proteobacteria | Alphaproteobacteria | Rhodospirillales | Acetobacteraceae | 12 |
|  | OTU1521 | Proteobacteria | Alphaproteobacteria | Rhodospirillales | Acetobacteraceae | 21 |
|  | OTU1515 | Proteobacteria | Alphaproteobacteria | Rickettsiales | Rickettsiales_Incertae_Sedis | 9 |
|  | OTU1528 | Proteobacteria | Alphaproteobacteria | Rickettsiales | Holosporaceae | 3 |
|  | OTU1632 | Proteobacteria | Alphaproteobacteria | Rickettsiales | Rickettsiaceae | 20 |
|  | OTU1587 | Proteobacteria | Alphaproteobacteria | Rickettsiales | unclassified_o__Rickettsiales | 24 |
|  | OTU1535 | Proteobacteria | Betaproteobacteria | Burkholderiales | Burkholderiaceae | 78 |
|  | OTU1544 | Proteobacteria | Betaproteobacteria | unclassified_c__Betaproteobacteria | unclassified_c__Betaproteobacteria | 21 |
|  | OTU1633 | Proteobacteria | Deltaproteobacteria | Bdellovibrionales | Bdellovibrionaceae | 49 |
|  | OTU1548 | Proteobacteria | Deltaproteobacteria | Bdellovibrionales | Bdellovibrionaceae | 3 |
|  | OTU1615 | Proteobacteria | Deltaproteobacteria | Myxococcales | mle1-27 | 46 |
|  | OTU1614 | Proteobacteria | Deltaproteobacteria | Myxococcales | mle1-27 | 30 |
|  | OTU582 | Proteobacteria | Deltaproteobacteria | Myxococcales | P3OB-42 | 4 |
|  | OTU1496 | Proteobacteria | Deltaproteobacteria | Myxococcales | Sandaracinaceae | 6 |
|  | OTU1494 | Proteobacteria | Deltaproteobacteria | Myxococcales | Phaselicystidaceae | 34 |
|  | OTU1634 | Proteobacteria | Deltaproteobacteria | Myxococcales | Sandaracinaceae | 6 |
|  | OTU1542 | Proteobacteria | Deltaproteobacteria | Myxococcales | Sandaracinaceae | 29 |
|  | OTU1676 | Proteobacteria | Deltaproteobacteria | Oligoflexales | 0319-6G20 | 112 |
|  | OTU1682 | Proteobacteria | Deltaproteobacteria | SAR324_clade_Marine_group_B_ | norank_o__SAR324_clade_Marine_group_B_ | 20 |
|  | OTU1527 | Proteobacteria | Deltaproteobacteria | SAR324_clade_Marine_group_B_ | norank_o__SAR324_clade_Marine_group_B_ | 24 |
|  | OTU1520 | Proteobacteria | Deltaproteobacteria | SAR324_clade_Marine_group_B_ | norank_o__SAR324_clade_Marine_group_B_ | 4 |
|  | OTU1665 | Proteobacteria | Deltaproteobacteria | unclassified_c__Deltaproteobacteria | unclassified_c__Deltaproteobacteria | 18 |
|  | OTU1674 | Proteobacteria | Gammaproteobacteria | Legionellales | Legionellaceae | 3 |
|  | OTU1493 | Proteobacteria | Gammaproteobacteria | Methylococcales | Methylococcaceae | 35 |
|  | OTU1529 | Proteobacteria | Gammaproteobacteria | Xanthomonadales | norank_o__Xanthomonadales | 8 |
|  | OTU1672 | Proteobacteria | unclassified_p__Proteobacteria | unclassified_p__Proteobacteria | unclassified_p__Proteobacteria | 39 |
|  | OTU1597 | Proteobacteria | unclassified_p__Proteobacteria | unclassified_p__Proteobacteria | unclassified_p__Proteobacteria | 14 |
|  | OTU1642 | Saccharibacteria | norank_p__Saccharibacteria | norank_p__Saccharibacteria | norank_p__Saccharibacteria | 34 |
|  | OTU1499 | TM6__Dependentiae_ | norank_p__TM6__Dependentiae_ | norank_p__TM6__Dependentiae_ | norank_p__TM6__Dependentiae_ | 6 |
|  | OTU1638 | unclassified_k__norank | unclassified_k__norank | unclassified_k__norank | unclassified_k__norank | 4 |
|  | OTU1603 | unclassified_k__norank | unclassified_k__norank | unclassified_k__norank | unclassified_k__norank | 42 |
|  | OTU1569 | Verrucomicrobia | Verrucomicrobiae | Verrucomicrobiales | Verrucomicrobiaceae | 76 |
|  | OTU1543 | Verrucomicrobia | Verrucomicrobiae | Verrucomicrobiales | Verrucomicrobiaceae | 23 |
|  | OTU1502 | Verrucomicrobia | Verrucomicrobiae | Verrucomicrobiales | Verrucomicrobiaceae | 33 |
|  | OTU1546 | Verrucomicrobia | Opitutae | Opitutae_vadinHA64 | norank_o__Opitutae_vadinHA64 | 78 |
| MYH | OTU1381 | Bacteroidetes | Sphingobacteriia | Sphingobacteriales | Sphingobacteriaceae | 52 |
|  | OTU1298 | Bacteroidetes | Sphingobacteriia | Sphingobacteriales | Saprospiraceae | 35 |
|  | OTU1301 | Bacteroidetes | Cytophagia | Cytophagales | Cytophagaceae | 19 |
|  | OTU1335 | Cyanobacteria | Cyanobacteria | norank_c__Cyanobacteria | norank_c__Cyanobacteria | 13 |
|  | OTU1212 | Cyanobacteria | Cyanobacteria | norank_c__Cyanobacteria | norank_c__Cyanobacteria | 10 |
|  | OTU1227 | Planctomycetes | Phycisphaerae | Phycisphaerales | Phycisphaeraceae | 41 |
|  | OTU1279 | Proteobacteria | Alphaproteobacteria | Rickettsiales | Mitochondria | 8 |
|  | OTU1449 | Proteobacteria | Deltaproteobacteria | Myxococcales | Phaselicystidaceae | 10 |
|  | OTU1236 | Proteobacteria | Deltaproteobacteria | Myxococcales | Archangiaceae | 14 |
|  | OTU1211 | Proteobacteria | Deltaproteobacteria | Myxococcales | Archangiaceae | 6 |
|  | OTU1203 | Proteobacteria | Deltaproteobacteria | Bdellovibrionales | Bacteriovoracaceae | 20 |
|  | **OTU1315** | Proteobacteria | Gammaproteobacteria | Methylococcales | Methylococcaceae | **100** |
|  | OTU1259 | Proteobacteria | Gammaproteobacteria | Legionellales | Legionellaceae | 7 |
|  | OTU1255 | TM6__Dependentiae_ | norank_p__TM6__Dependentiae_ | norank_p__TM6__Dependentiae_ | norank_p__TM6__Dependentiae_ | 40 |
|  | OTU1241 | Verrucomicrobia | Spartobacteria | Chthoniobacterales | Chthoniobacteraceae | 41 |
| GH | OTU22 | Actinobacteria | Actinobacteria | Solirubrobacterales | TM146 | 79 |
|  | OTU128 | Actinobacteria | Actinobacteria | unclassified_c__Actinobacteria | unclassified_c__Actinobacteria | 391 |
|  | OTU132 | Bacteroidetes | Flavobacteriia | Flavobacteriales | Flavobacteriaceae | 20 |
|  | OTU212 | Bacteroidetes | Bacteroidetes_Incertae_Sedis | Order_III | CK06-06-Mud-MAS4B-21 | 6 |
|  | OTU65 | Bacteroidetes | Sphingobacteriia | Sphingobacteriales | NS11-12_marine_group | 20 |
|  | OTU130 | Bacteroidetes | Sphingobacteriia | Sphingobacteriales | NS11-12_marine_group | 115 |
|  | OTU12 | Bacteroidetes | Sphingobacteriia | Sphingobacteriales | Saprospiraceae | 760 |
|  | OTU46 | Chloroflexi | Chloroflexia | Chloroflexales | Chloroflexaceae | 132 |
|  | OTU23 | Cyanobacteria | Cyanobacteria | norank_c__Cyanobacteria | norank_c__Cyanobacteria | 433 |
|  | OTU18 | Cyanobacteria | Cyanobacteria | norank_c__Cyanobacteria | norank_c__Cyanobacteria | 11 |
|  | OTU21 | Firmicutes | Bacilli | Bacillales | Paenibacillaceae | 44 |
|  | OTU109 | Gemmatimonadetes | Gemmatimonadetes | Gemmatimonadales | Gemmatimonadaceae | 24 |
|  | OTU70 | Proteobacteria | Alphaproteobacteria | Caulobacterales | Hyphomonadaceae | 7 |
|  | OTU96 | Proteobacteria | Alphaproteobacteria | Rickettsiales | Rickettsiales_Incertae_Sedis | 142 |
|  | OTU125 | Proteobacteria | Alphaproteobacteria | Rickettsiales | unclassified_o__Rickettsiales | 20 |
|  | OTU121 | Proteobacteria | Alphaproteobacteria | Rickettsiales | Rickettsiaceae | 24 |
|  | **OTU17** | Proteobacteria | Betaproteobacteria | Burkholderiales | Alcaligenaceae | **7827** |
|  | OTU27 | Proteobacteria | Deltaproteobacteria | Oligoflexales | 0319-6G20 | 75 |
|  | OTU104 | Proteobacteria | Deltaproteobacteria | SAR324_clade_Marine_group_B_ | norank_o__SAR324_clade_Marine_group_B_ | 49 |
|  | OTU61 | unclassified_k__norank | unclassified_k__norank | unclassified_k__norank | unclassified_k__norank | 18 |
|  | OTU38 | unclassified_k__norank | unclassified_k__norank | unclassified_k__norank | unclassified_k__norank | 26 |

**TABLE S4** Spearman correlation between dominant indicator species and environmental parameters

| Factors | The top 5 indicator species with the highest abundance | | | | |
| --- | --- | --- | --- | --- | --- |
|  | Species 1 | Species 2 | Species 3 | Species 4 | Species 5 |
| T (℃) | -0.41 | -0.48 | -0.07 | -0.29 | 0.61* |
| pH | 0.51 | 0.43 | 0.63* | 0.54 | 0.50 |
| DO (mg/L) | -0.05 | -0.18 | 0.10 | 0.03 | 0.35 |
| EC (uS/cm) | 0.79** | 0.87** | 0.80** | 0.74** | 0.06 |
| SAL(ng/L) | -0.10 | -0.26 | -0.04 | -0.08 | 0.24 |
| TN (mg/L) | 0.78** | 0.91** | 0.83** | 0.74** | 0.12 |
| NO_3_^-^ (mg/L) | 0.83** | 0.84** | 0.86** | 0.71* | 0.31 |
| NO_2_^-^ (mg/L) | 0.80** | 0.51 | 0.56 | 0.76** | 0.45 |
| NH_4_^+^ (mg/L) | 0.73** | 0.89** | 0.80** | 0.71* | 0.11 |
| C/N | -0.32 | -0.53 | -0.38 | -0.31 | -0.06 |
| TC (mg/L) | 0.73** | 0.72** | 0.86** | 0.76** | 0.30 |
| IC (mg/L) | 0.10 | 0.33 | 0.47 | 0.19 | 0.24 |
| TOC (mg/L) | 0.84** | 0.76** | 0.84** | 0.83** | 0.32 |
| SO_4_^2-^ (mg/L) | 0.22 | 0.12 | 0.30 | 0.21 | 0.32 |
| PO_4_^3-^ (mg/L) | 0.17 | 0.21 | 0.09 | 0.04 | 0.19 |

TABLE S5 Interactive-forward-selection results in Redundancy analysis

| Entire bacterial community | | | | Shared taxa | | | | Unique taxa | | | |
| --- | --- | --- | --- | --- | --- | --- | --- | --- | --- | --- | --- |
| Name | Explains % | F | P | Name | Explains % | F | P | Name | Explains % | F | P |
| TOC | 52.4 | 11.0 | 0.002 | EC | 56.1 | 12.8 | 0.002 | TOC | 43.9 | 7.8 | 0.002 |
| IC | 15.9 | 5.2 | 0.002 | pH | 11.2 | 3.1 | 0.012 | PCNM1 | 32.4 | 12.3 | 0.002 |
| PCNM1 | 9.9 | 2.4 | 0.042 | SAL | 7.4 | 1.9 | 0.045 | IC | 7.6 | 3.7 | 0.042 |
| pH | 6.2 | 2.3 | 0.034 | - | - | - | - | - | - | - | - |

**
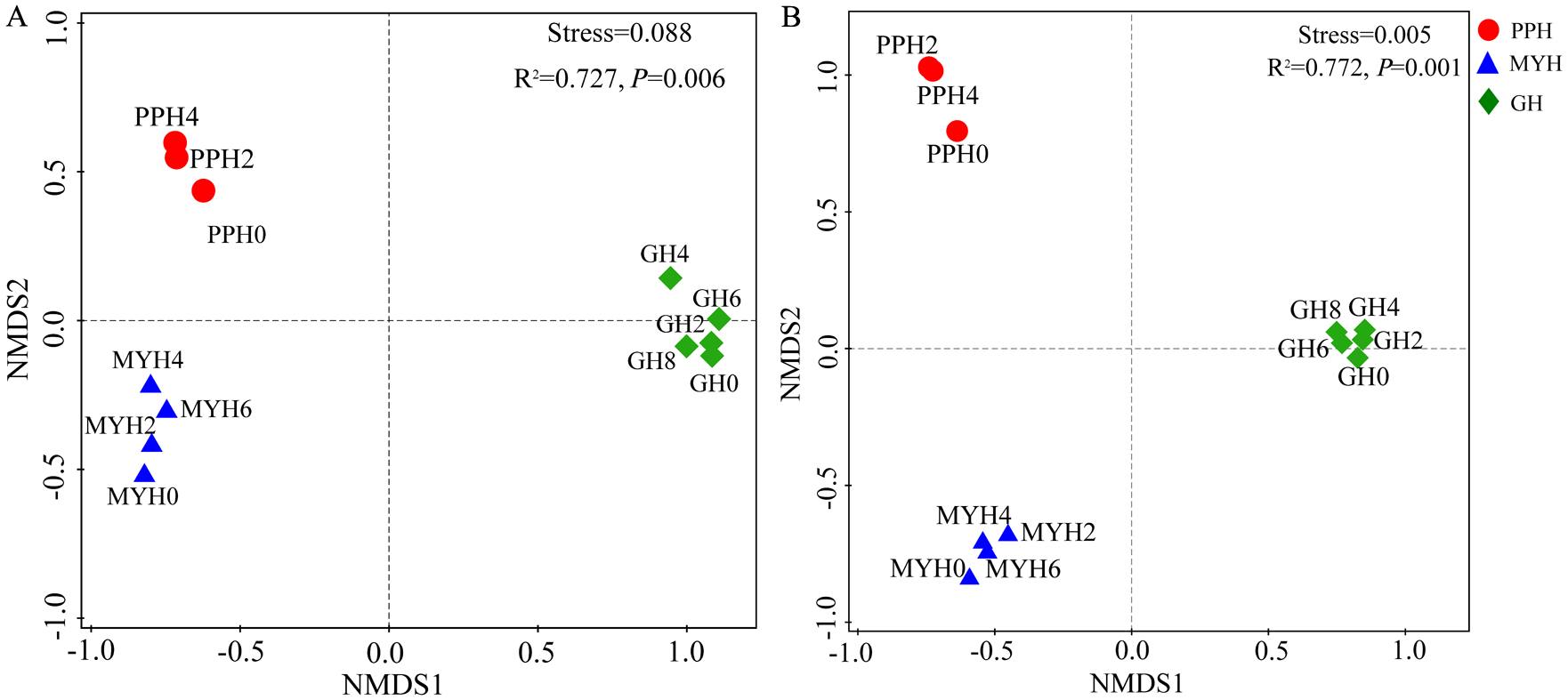
**

**FIG S1** Shared taxa (A) and unique taxa(B) structures analysis shown as non-metric multidimensional scaling (NMDS) plots based on Bray-Curtis distance (on OTU level) for pairwise differences between datasets originating from the PPH, MYH and GH.
